# Peer-delivered harm reduction and recovery support services: initial evaluation from a hybrid recovery community drop-in center and syringe exchange program

**DOI:** 10.1186/s12954-018-0258-2

**Published:** 2018-10-22

**Authors:** Robert D. Ashford, Brenda Curtis, Austin M. Brown

**Affiliations:** 1 Substance Use Disorders Institute, University of the Sciences, 2111, Philadelphia, PA 19131 USA; 2 Center on the Continuum of Care in the Addictions, Psychiatry - Addictions, University of Pennsylvania, Philadelphia, PA 19131 USA; 3 Center for Young Adult Addiction and Recovery, Kennesaw State University, Kennesaw, GA 30144 USA

**Keywords:** Harm reduction, Syringe exchange, Peer recovery support services, Recovery community organizations, Intravenous substance use

## Abstract

**Background:**

Recovery from substance use disorder (SUD) is often considered at odds with harm reduction strategies. More recently, harm reduction has been categorized as both a pathway to recovery and a series of services to reduce the harmful consequences of substance use. Peer recovery support services (PRSS) are effective in improving SUD outcomes, as well as improving the engagement and effectiveness of harm reduction programs.

**Methods:**

This study provides an initial evaluation of a hybrid recovery community organization providing PRSS as well as peer-based harm reduction services via a syringe exchange program. Administrative data collected during normal operations of the Missouri Network for Opiate Reform and Recovery were analyzed using Pearson chi-square tests and Monte Carlo chi-square tests.

**Results:**

Intravenous substance-using participants (*N* = 417) had an average of 2.14 engagements (SD = 2.59) with the program. Over the evaluation period, a range of 5345–8995 sterile syringes were provided, with a range of 600–1530 used syringes collected. Participant housing status, criminal justice status, and previous health diagnosis were all significantly related to whether they had multiple engagements.

**Conclusions:**

Results suggest that recovery community organizations are well situated and staffed to also provide harm reduction services, such as syringe exchange programs. Given the relationship between engagement and participant housing, criminal justice status, and previous health diagnosis, recommendations for service delivery include additional education and outreach for homeless, justice-involved, LatinX, and LGBTQ+ identifying individuals.

## Background

Peer-based recovery community organizations (RCO) and programs have presented unique opportunities for the study of community responses to the opioid crisis in the USA. In the last decade, the number of RCOs in the USA has expanded in large part due to federal investment through the Recovery Community Services Program (RCSP) and Access to Recovery (ATR) Substance Abuse and Mental Health Services Administration (SAMHSA) grants [1]. Outside of the USA, peer-based harm reduction programs have shown that peer-delivered services are a viable option in reducing death and transmission of disease and having a positive impact on the quality of life of people who use drugs (PWUD) and those seeking recovery [2–4]. As the USA continues to expand its understanding and acceptance of harm reduction practices, RCOs can adapt to embrace both recovery and harm reduction strategies to play an even more important role in communities impacted the most by opioids and other substance use.

### Peer-based recovery community organizations

RCOs are non-profit, non-governmental organizations which are directly led by members of recovery community (i.e., peers in recovery from substance use disorders) [5]. The organizations provide recovery support services, advocacy, and community education around substance use disorder (SUD). RCOs are not only led by peers, but peers also deliver the peer recovery support services (PRSS) that are delivered programmatically; PRSS encompass peer recovery coaching, consumer advocacy, mentoring, and facilitation of vocational groups focused on employment, housing, and education [6]. RCOs have emerged as a promising new innovation driven by social need, created by the recovery community, and grown by and for the populations they serve [7, 8]. From a service standpoint, in addition to providing recovery-specific brick and mortar space for pro-social activities and delivery of PRSS, RCOs also provide community outreach and operate as an information and referral clearinghouse for other SUD and non-SUD support organizations such as local housing, local treatment providers, and other social support services [6].

PRSS are delivered in a variety of settings, including RCOs [9], emergency departments [10], collegiate recovery programs [11], and in general community locations [12]. In a recent systematic review, Bassuk and colleagues report that PRSS have a positive impact on participants and substance use disorder (SUD) outcomes [13]. The study of PRSS remains in its infancy, and additional evaluation of the mechanism of action and long-term effects is still needed. However, preliminary evidence suggests that the use of peers in the recovery process can lead to reductions in hospital readmissions and length of sobriety, post-discharge treatment plan adherence, increased housing stability, and improvements in mental health functioning [13].

### Peer-based harm reduction strategies

Reflexively centered around recovery initiation and sustainment, a missing element in the framework of peer-delivered recovery supports, especially in RCOs, is the capacity to accommodate and integrate harm reduction strategies. Harm reduction initiatives—such as syringe exchange programs (SEP) and drug consumption rooms—have shown reductions in the sharing of syringes [2], decreases in HIV infection rates [3], and reductions in overdose deaths [4]. While many harm reduction initiatives are delivered in a traditional, professionalized manner, there are also peer-delivered harm reduction strategies that mirror the nature of PRSS [14–17].

Internationally, peers have been used to engage people who use drugs (PWUDs) in an effort to connect to clinical services treatment and recovery supports, but also to reduce the harms associated with active substance use. For example, a peer-based SEP in Vancouver increased the service reach of the SEP to individuals not normally engaged by proto-typical programs [15]. More recently, improved mental health outcomes have been associated with the use of peer-based SEPs. For example, Hay and colleagues reported that PWUD who engaged with peer-based SEPs reported lower levels of depression and anxiety and reported higher levels of life satisfaction, compared to PWUD who engaged with non-peer-based SEPs [16]. More importantly, the rate of health information exchange was greater for peer-based SEPs than non-peer SEPs [16]. As RCOs have a robust peer workforce at the ready, the opportunity to bridge into provision of harm reduction services may be possible.

**Fig. 1:**
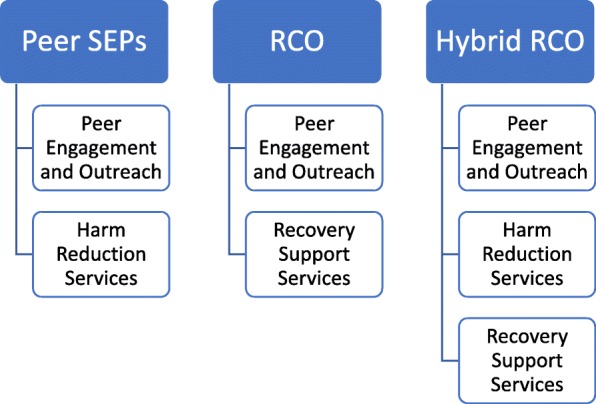
Peer SEP, RCO, and hybrid RCO comparison models. SEP = syringe exchange program; RCO = recovery community organization

For example, RCOs have a primary interest in the initiation of recovery and this is an area where harm reduction strategies have seen success. Previous evaluation of SEPs has found that the successful referral of participants to SUD treatment is as high as 74% [18]. Additionally, of those referred, over 80% also remained engaged in treatment for at least 90 days. Though SEPs were designed to decrease the risk of disease transmission—which they are also successful with [19]—the programs also engage participants in ancillary services at rates that cannot be understated. With a plausible peer workforce and a synergy in desired outcomes, it stands to reason then that a hybrid model of RCO and peer-based SEP (Fig. 1) may be an effective and innovative intervention.

### Recovery community organizations providing harm reduction services

The current study seeks to gain a better of the understanding of the characteristics of an RCO providing peer-based SEP services (i.e., hybrid RCO model) through a preliminary evaluation of the first known program in the USA—the Missouri Safe Project (MO Safe) of the Missouri Network for Opiate Reform and Recovery (MO Network). As of the writing of this paper, the MO Safe project is the only RCO that officially provides an SEP component.

We use a cross-sectional design to quantify the frequency of engagement (i.e., number of times served by the program) and the descriptive characteristics of participants of this hybrid program—including HIV and hepatitis C (HCV), housing, and criminal justice status. The primary research hypothesis is that consumers will engage multiple times with services delivered by the hybrid RCO, with those consumers who have HIV and HCV engaging more frequently due to the added provision of syringe exchange services. Additionally, by determining the frequency of engagements and demographics of those served, it will be possible to better identify and design engagement strategies to initiate recovery and reduce harm via future hybrid RCOs. Given the infancy of the science involving RCOs and PRSS, one scientific imperative is to lay the preliminary groundwork for future study by surveying the first known program to offer both peer-based recovery supports and harm reduction services. We believe that by reporting the various taxonomy of RCO and harm reduction services, along with who utilizes such services when delivered in concert, future study may be aimed at establishing the ongoing efficacy of such organizations and outcomes for those involved.

In summary, RCOs are continuing to expand throughout the country and peer support models are emerging in various permutations from collegiate recovery programs to crisis point deployment such as in emergency room settings for post-drug poisoning emergencies (i.e., overdose). It is clear that peer-based services and RCOs will continue to grow as frontline recovery access and sustainment mechanisms. The study of harm reduction strategies as a component of RCO and peer-based services, such as SEPs, may open up a crucial area for the expansion of the service mission of RCOs nationwide and help design strategies for engagement of PWUD.

## Methods

### Missouri Network for Opiate Reform and Recovery: Missouri Safe Project

The Missouri Network for Opiate Reform and Recovery (MO Network) is an RCO established in 2015 that provides PRSS via a drop-in recovery center and mobile outreach unit, recovery residences, naloxone distribution, and overdose education, as well as testing for HIV and HCV. As part of the organization’s ongoing efforts to respond to the opioid epidemic in Missouri, a partnership was formed with Criminal Justice Ministry in 2016 to begin offering syringe exchange services in a comprehensive manner. Along with the services offered through MO Network, the new project (Missouri Safe Project; MO Safe) began offering syringe exchange (with no requirement to exchange used syringes in order to receive sterile syringes, in accordance with international best practice recommendations), wound care, sterile supplies, and basic hygiene products. MO Network and MO Safe services are provided by peers and both offered at the drop-in center as well as via the mobile outreach unit.

### Recruitment

Recruitment was completed as part of regular operations of the MO SAFE project; we analyzed administrative data collected by project staff for the selected time period and did not complete any data collection in addition to the administrative data provided by project staff. During February, March, and April 2018, participants were seen either at the MO Network recovery drop-in center on a walk-in basis or via mobile outreach. In each engagement, a peer specialist spoke with participants to discuss available services and resources as part of MO Safe and the additional PRSS available via MO Network. All data analyzed for this study were derived from the participant’s file, which was comprised of data collected and entered by the peer specialists from initial and follow-up engagements. Participants provided consent that data derived from the engagement could be used for future program evaluation. Following IRB review at Kennesaw State University, the study was indicated as exempt as it made use of de-identified data that was collected for peer service delivery purposes.

### Measures

Data from this study were identified and collected by MO Network staff prior to the plans for this study. Participants provided basic demographic information (self-reported age, gender, race/ethnicity, sexual orientation), as well as self-reported information related to housing status, criminal justice involvement, HIV and HCV status, and any prior health diagnoses given to them by a medical professional. Additionally, participants reported whether naloxone had been administered to them between peer engagements and the number of sterile syringes received and used syringes returned; the number of syringes exchanged is reported as a range due to the manner in which data was collected by the program staff. Program staff also recorded dates of peer engagement for each participant with a unique identifier code, which we used to create a peer engagement variable. In order to identify variables associated with participants not returning after an initial engagement, participants with only one unique engagement record were denoted as “single engagement” and all participants with multiple unique engagement records were denoted as “multiple engagement.”

### Data analysis

All data was analyzed with SPSS V24.0. Descriptive statistics were analyzed for all participants. Following the initial descriptive analysis, it was determined that ad hoc testing of demographic and outcomes variables (e.g., Narcan administration, syringes received and returned, single or multiple peer engagements) would be used to identify any significant relationships. Initially, Pearson chi-square tests were proposed; however, when analyzing cross tabulations, it was determined that tables had more than 20% of cells with a less than expected 5-count. Thus, Monte Carlo testing [20, 21] was used when the cell count requirement was not satisfied. All Monte Carlo tests were performed using 100,000 samples, random starting seed, and a 99% confidence interval (CI). Results from each Monte Carlo test are reported using the chi-square likelihood ratio statistic, degrees of freedom (DF), simulated exact *p* value, and the 99% confidence interval (*X*^2^ (DF, *N*) = L.R statistic value, *p* value, (99% CI LL, 99% CI UL).

## Results

### Participants

**Fig. 2:**
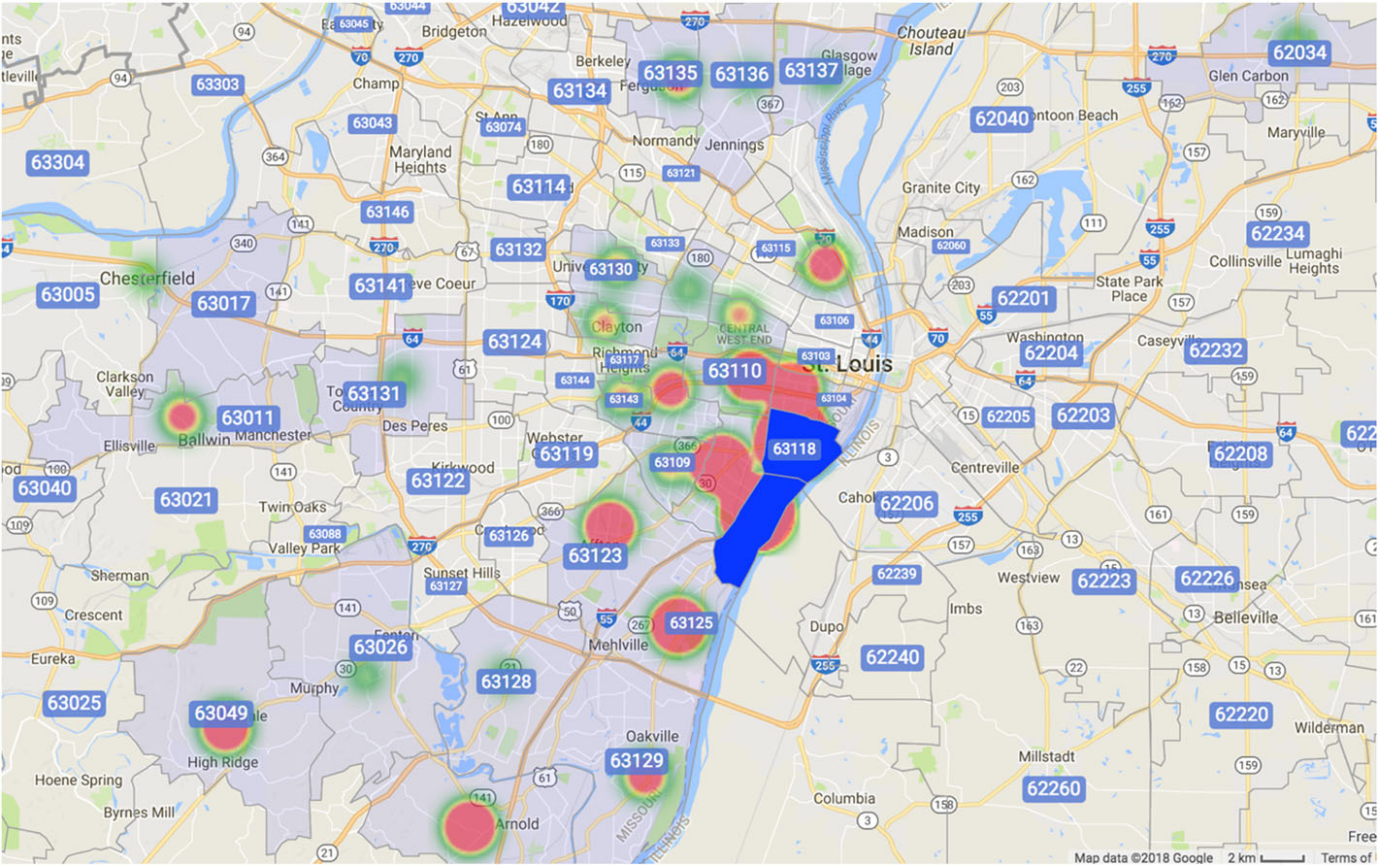
Zip code heat map of intravenous substance use of participants

Participants (*N* = 417) had a mean age of 35.59 years (SD = 10.42), with the majority being male (58.5%), white/Caucasian (66.9%), and heterosexual (84.9%). All participants (100%) were intravenous substance users. More than half of participants reported stable housing (55.9%). A large portion of participants reported having HCV (34.1%), while a smaller percentage reporting having HIV (3.1%). Less than half of participants had either previously been on, or were currently on, probation or parole (38.1%). Only a small number of participants had co-occurring health concerns, with 13.4% reporting a previous mental health illness. Participants reported intravenous substance use in multiple zip codes surrounding St. Louis (Fig. 2); however, 41.9% of all reported use was in zip codes in, or adjacent to, the physical location of MO Network’s drop-in center. Full participant demographics are available in Table 1.

**Table 1:** Participant characteristics

	(*n* = 417)	
	*n*	(%)
Age (years) *M* = 35.59, SD = 10.42		
Gender		
Male	244	(58.5)
Female	166	(39.8)
Other	7	(1.7)
Race/ethnicity		
White	279	(66.9)
Black	99	(23.7)
Multi-racial	29	(7.0)
Other	10	(2.4)
Sexual orientation		
Heterosexual	354	(84.9)
Bisexual	32	(7.7)
Homosexual	31	(7.5)
Housing status		
Stable housing	233	(55.9)
Homeless	92	(22.1)
Transitional housing	11	(2.6)
Couch surfing	62	(14.9)
Unknown	19	(4.6)
HIV status		
Positive	13	(3.1)
HCV status		
Positive	142	(34.1)
Probation/parole status		
Currently on	36	(8.6)
Previously on	123	(29.5)
Medical diagnosis		
Mental health diagnosis	56	(13.4)
Diabetes	7	(1.7)
Infection	13	(3.1)
Multiple	14	(3.4)
Zip codes of highest use		
63111	71	(17.0)
63118	76	(18.2)
63116	28	(6.7)
Multiple engagements at RCO		
Yes	149	(35.7)
Total number of participants (*n* = 417) Total engagements = 895, *M* = 2.14, SD = 2.59		
Total Narcan dispensed		
IM	9	–
Nasal	99	–
Narcan administered*		
Yes and 911 called	90	(6.8)
Yes and 911 not called	61	(10.1)
Sterile syringes dispensed		
Range 5345–8955	–	–
Used syringes received		
Range 600–1530	–	–

* *n* = 895 as result is calculated from all engagements with duplicate participants

### Naloxone administration, syringe exchange, and peer engagement

All participants engaging in the MO Safe program are current intravenous substance users. Overall, 895 total peer engagements with 417 unduplicated participants occurred during the study period (peer engagements: *M* = 2.14, SD = 2.59). Slightly more than a third of all participants had multiple engagements (35.7%, *N* = 149). Of the 895 total engagements, participants reported 151 instances (16.9%) of naloxone (e.g., brand name: Narcan) administration since their last engagement. Additionally, 108 naloxone kits were given out to participants. Over the course of the study period, the MO Safe program dispensed a range of 5345–8995 sterile syringes, while a range of 600–1530 used syringes were collected in the same period. All participants (*N* = 417) received and returned at least 1 sterile and 1 used syringe. Results from all significant Monte Carlo chi-square tests are available in Table 2.

**Table 2:** Significant Monte Carlo chi-square test results from multiple participant variable pairs

Variable pair	df	*N*	*X* ^2^	*p*	99% CI (LL, UL)
Multiple peer engagements and housing status	4	417	9.451	.05	(.049, .051)
Multiple peer engagements and probation/parole status	3	417	30.194	< .001	(.000, .000)
Multiple peer engagements and previous health Dx	6	417	32.485	< .001	(.000, .000)
Multiple peer engagements and race/ethnicity	4	417	12.406	.011	(.010, .012)
Naloxone administration and probation/parole status	2	417	22.693	.007	(.007, .008)
Naloxone administration and sexual orientation	9	417	25.347	.018	(.017, .019)
Syringes received and HIV status	4	417	17.013	.024	(.022, .025)
Syringes returned and HIV status	6	417	14.042	.04	(.039, .042)

*df* degrees of freedom, *CI* confidence interval, *LL* lower limit, *UL* upper limit, *Dx* diagnosis

### Variance among participant health, housing, and criminal justice variables

Monte Carlo chi-square tests were used to analyze relationships between various descriptive variables and outcomes variables.

#### Multiple peer engagements and housing status

Results suggest that the relationship between having multiple peer engagements and participant housing status was significant (*X*^2^ (4, *N* = 417) = 9.451, *p* = .050 (99% CI, .049, .051). Participants reporting homelessness were least likely to have multiple engagements compared to clients reporting stable housing, transitional housing, or living doubled up. Also, of note is that participants living doubled up were most likely to have multiple engagements than any other reported housing status.

#### Multiple peer engagements and probation/parole status

Results suggest that the relationship between having multiple peer engagements and participant probation or parole status was significant (*X*^2^ (3, *N* = 417) = 30.194, *p* < .001 (99% CI, .000, .000). Participants who are currently on probation or parole were least likely to have multiple engagements, while those who were not currently on probation/parole but had been previously were most likely to have multiple engagements.

#### Multiple peer engagements and previous health diagnosis

Results suggest that the relationship between having multiple peer engagements and previous health diagnosis was significant (*X*^2^ (6, *N* = 417) = 32.485, *p* < .001 (99% CI, .000, .000). Participants that have had an infection, such as endocarditis, or have had previous mental health diagnosis are least likely to have multiple engagements, while those with a previous diabetes diagnosis are most likely to have multiple engagements.

#### Naloxone administration and probation/parole status

Results suggest that the relationship between naloxone administration to the participant since the last peer engagement and participant probation or parole status was significant (*X*^2^ (2, *N* = 417) = 22.693, *p* = .007 (99% CI, .007, .008). Participants not currently on probation/parole but had been previously were least likely to have had naloxone administered since the last peer engagement, while participants currently on probation and parole were most likely to have had naloxone administered since the last peer engagement.

#### Syringes received and HIV status

Results suggest that the relationship between the number of syringes given to the participant and participant HIV status was significant (*X*^2^ (4, *N* = 417) = 17.013, *p* = .024 (99% CI, .022, .025). Participants who have positive HIV status were most likely to receive the highest range of syringes (11–20 syringes) compared to those who had a negative HIV status.

#### Syringes returned and HIV status

Results suggest that the relationship between the number of syringes returned by the participant and participant HIV status was significant (*X*^2^ (6, *N* = 417) = 14.042, *p* = .040 (99% CI, .039, .042). Participants who have positive HIV status were most likely to return all ranges of syringes (1–5, 6–11, 11–20 syringes) than participants who have a negative HIV status.

#### Non-significant relationships

Results for the following variables were not significant (*p* > .05) and suggest that there is no significant relationship between the variables: (a) multiple peer engagements and HIV or HCV status; (b) naloxone administration and HIV or HCV status; (c) naloxone administration and housing status; (d) syringes received and HCV status, housing status, probation/parole status, or previous health diagnosis; and (e) syringes returned and HCV status, housing status, probation/parole status, or previous health diagnosis.

### Variance among gender, race/ethnicity, and sexual orientation variables

Monte Carlo chi-square tests were also used to analyze relationships between various descriptive variables and outcomes variables.

#### Multiple peer engagements and race/ethnicity

Results suggest that the relationship between having multiple peer engagements and participant race/ethnicity was significant (*X*^2^ (4, *N* = 417) = 12.406, *p* = .011 (99% CI, .010, .012). Participants self-reporting as LatinX (i.e., male and female Latin-identifying individuals; LatinX is used as a gender-neutral term here) are least likely to have multiple engagements, while participants self-reporting as multiracial are most likely to have multiple engagements.

#### Naloxone administration and sexual orientation

Results suggest that the relationship between naloxone administration to the participant since the last peer engagement and participant sexual orientation was significant (*X*^2^ (9, *N* = 417) = 25.347, *p* = .018 (99% CI, .017, .019). Participants who identified as bisexual were most likely to have naloxone administered since the last peer engagement, while homosexual clients were least likely to have naloxone administered since the last peer engagement.

#### Non-significant relationships

Results for the following variables were not significant (*p* > .05) and suggest that there is no significant relationship between the variables: (a) multiple peer engagements and gender or sexual orientation and (b) naloxone administration and race or gender.

## Discussion

Innovative, community-based programs are emerging across the USA as a response to the opioid crisis. Many of these programs are peer-based and provide a valuable addition to the strained SUD treatment infrastructure in the country [22]. Combining RCOs with harm-reduction interventions, such as the MO Safe project, is one such innovation that shows potential. RCOs have traditionally provided PRSS and treatment referrals [23]. However, recently, they have begun partnering with other entities like primary health care and emergency departments to provide initial engagement and referral for patients experiencing an accidental drug poisoning (i.e., an overdose) [24]. Given the use of peers and RCO primary goals of initiating and sustaining recovery, RCOs are well positioned to expand their menu of services to include harm reduction. Providing harm reduction services as a supplement to recovery supports allows for engagement of individuals who are at risk of disease, such as HCV and HIV, and overdose. At-risk populations have been found to engage at higher rates with peer-based initiatives [25, 26]. Additionally, for the subset of this at-risk population that is interested in treatment initiation, the close proximity to recovery supports and treatment referrals provides a distinct opportunity. The current study provides preliminary evidence that a hybrid RCO/harm reduction model is feasible and effective at engaging people who inject drugs.

Previous research has reported that individuals who struggle with homelessness are at high risk for overdose [27]. Results from this study indicate that those without stable housing were least likely to engage multiple times. As a particularly vulnerable population, expanding outreach from RCOs to homelessness encampments or mobile outreach teams could better engage individuals without stable housing and at elevated risk for overdose.

Clients currently involved with the criminal justice system, especially those recently released from a criminal justice facility, are also at higher risk for overdose [28, 29]. We found that participants currently on probation or parole were least likely to have multiple engagements and were the most likely to have had naloxone administered since their last engagement. RCOs have the capacity to engage individuals multiple times, and this should be a priority among individuals currently on probation and parole, given the relationship to increased naloxone administration. Forming partnerships with local probation and parole professionals may increase engagement. It is possible that the stigma and discrimination PWUD often experience via criminal justice professionals is related to the lack of multiple engagements [30, 31] and should be further explored and mediated given the elevated risk. Recent innovations highlighting partnerships between the criminal justice system such as the Police Assisted Addiction and Recovery Initiative (PAARI) [32, 33] could serve as models for future partnerships.

Results from the current study indicate that individuals with previous infections are least likely to have multiple engagements with peer specialists. Increased education regarding the potential benefits of continuing to receive harm reduction services such as SEP or sterile medical supplies may be warranted for this population. HCV and HIV status were not significantly related to having multiple engagements, suggesting that services are perceived as beneficial to participants regardless of HCV or HIV status. Participants that were positive for HIV were most likely to receive a greater number of sterile syringes, as well as to return all ranges of used syringes. This finding is supportive of the continued use of SEPs. Increasing the distribution of sterile syringes and reclamation of used syringes is associated with a decline in the transference of communicable disease, though more research needs to be conducted, since multiple confounding variables may impact the study of SEP efficacy [34]. However, the cost-benefit of such harm reduction strategies has been established [35].

The results highlight two other vulnerable populations who may benefit from additional support. First, LatinX participants were least likely to have multiple engagements. Relatable peers with culturally specific knowledge should be used to increase culturally congruent services. LatinX populations are at elevated risk for overdose and HIV/HCV, and it is important to ensure continuous use of services however possible. Second, bisexual participants were most likely to have had naloxone administered since the last engagement. Previous research has reported similar findings [36], suggesting that bisexual participants may benefit from additional overdose prevention education and assured naloxone distribution.

### Limitations

Results from the current study should be viewed in light of the following limitations. Given the transient and often-criminalized status of the participants, self-report measures may be inaccurate, under-reported, or misreported. Secondly, as the research team was not involved in the data collection and management protocol, many potentially beneficial variables and data points were not collected. For example, the current evaluation did not account for which PRSS were used by participants, which brings into question the efficacy of offering recovery supports in addition to harm reduction services or if utilization of other services minimizes the desire for multiple peer specialist engagements. Data about syringes that were exchanged and distributed also suffers a limiting factor in this way, as the program staff collected this data via a wide range that only allowed for a wide variance range in the results of the current study. Similarly, while it was reported that all participants were intravenous substance users, the duration and severity of all past substance use and all types of substances used intravenously are unknown. Finally, though the sample of the current study was large, it was isolated to one small geographic area in the USA. As such, the results are not generalizable to all populations.

### Future directions

This study highlights the importance of data collection and identifications for improved services in both RCO operations and in harm reduction strategies. RCOs, especially those that provide harm reduction services, should seek to collect such data, identify areas of vulnerability, and adjust services to better respond to the needs of vulnerable clients. While RCOs are still in their infancy, service delivery standards, identifying community needs, and seeking more effective means of engaging clients should be a priority of such organizations. The current study provides evidence that RCOs can successfully integrate harm reduction services with concurrent PRSS offerings. The fusion of recovery supports and individual engagement through harm reduction may increase the chances that those with an ongoing SUD remain connected to a community of recovery and that such an organization can help safeguard health and wellbeing even with clients who may not be seeking recovery at the time. RCOs and hybrid RCOs are a unique area for future studies based in community- and practice-based evidence with roots in action and participatory research.

More broadly, collaborative community inquiry and action/participatory methodologies should be utilized in future studies to establish practice-based evidence for research and service design [37–39]. Collaborative inquiry and methodologies are fundamental to community-based practice, research, and service delivery. The engagement of vulnerable populations should be formulated to simultaneously identify needs and build service delivery methods through such inquiry [40, 41]. The use of collaborative approaches can also help to address potential limitations of data collection, such as experienced in the current study, as partnerships between community-based organizations and research institutions can devise manners to cost-effectively and efficiently collect key data.

One strength of this study is in providing a roadmap for ongoing identification of vulnerabilities in service delivery. Such analysis should be a contingency in the organizing and planning of services through community assets such as RCOs. And finally, empirical assessment of service impact should be closely aligned with subjective client experience and placed within a larger ecological theory as a general research framework. Future research into RCOs, harm reduction strategies, and community-based recovery support should seek to incorporate such mixed methods in order to best capture community impact, user experience, and recovery phenomena in order to scientifically identify opportunities to sustain life and stabilize health while helping to initiate and sustain SUD recovery.

## Conclusion

RCOs are well-equipped to provide expanded services bridging into the harm reduction arena. Peer-based services increase engagement of people with SUD or that use substances regularly. RCOs that provide PRSS and harm reduction services should work to identify specific populations at increased risk of overdose and disease transmission and seek to address such populations with targeted and tiered responses that begin with harm reduction strategies, education, and prevention while offering recovery services when applicable.

Evidence from this study suggests that syringe exchange programs (SEPs) embedded within RCOs provide an additional method for reducing risk of infection, providing valuable resources, and increasing engagement with vulnerable populations. The benefit of SEPs and the cost-effectiveness of such programs are well established and should be considered a fundamental best practice in the harm reduction tool kit. Moving forward, it is also worthwhile to begin considering adding these programs as basic services in all RCOs.
